# Ethnic differences in alcohol and drug use and related sexual risks for HIV among vulnerable women in Cape Town, South Africa: implications for interventions

**DOI:** 10.1186/1471-2458-13-174

**Published:** 2013-02-26

**Authors:** Bronwyn Myers, Tracy L Kline, Felicia A Browne, Tara Carney, Charles Parry, Kim Johnson, Wendee M Wechsberg

**Affiliations:** 1 Alcohol and Drug Abuse Research Unit, South African Medical Research Council, PO Box 19070, Tygerberg 7505, South Africa; 2 Department of Psychiatry and Mental Health, University of Cape Town, Cape Town, South Africa; 3 RTI International, 3040 Cornwallis Road, Research Triangle Park, NC 27709, USA; 4 Harvard School of Public Health, Harvard University, 677 Huntington Ave., Boston, MA, USA; 5 Department of Psychiatry, Stellenbosch University, Stellenbosch, South Africa; 6 Health, Policy and Administration, Gillings School of Global Public Health, The University of North Carolina, Chapel Hill, NC, USA; 7 Psychology in the Public Interest, North Carolina State University, Raleigh, NC, USA; 8 Psychiatry and Behavioral Sciences, Duke University School of Medicine, Durham, NC, USA

**Keywords:** Ethnic differences, Alcohol and other drugs, Sexual risks, Women, South Africa

## Abstract

**Background:**

Alcohol and other drug (AOD) use among poor Black African and Coloured women in South Africa compounds their sexual risk for HIV. Given South Africa’s history of ethnic disparities, ethnic differences in sex risk profiles may exist that should be taken into account when planning HIV risk reduction interventions. This paper aims to describe ethnic differences in AOD use and AOD-related sexual risks for HIV among vulnerable women from Cape Town, South Africa.

**Method:**

Cross-sectional data on 720 AOD-using women (324 Black African; 396 Coloured) recruited from poor communities in Cape Town were examined for ethnic differences in AOD use and AOD-related sexual risk behavior.

**Results:**

Ethnic differences in patterns of AOD use were found; with self-reported drug problems, heavy episodic drinking and methamphetamine use being most prevalent among Coloured women and cannabis use being most likely among Black African women. However, more than half of Black African women reported drug-related problems and more than a third tested positive for recent methamphetamine use. More than a third of women reported being AOD-impaired and having unprotected sex during their last sexual encounter. Coloured women had four-fold greater odds of reporting that their last sexual episode was AOD-impaired and unprotected than Black African women. In addition, close to one in two women reported that their sexual partner was AOD-impaired at last sex, with Coloured women having three-fold greater odds of reporting that their partner was AOD-impaired at last sex than Black African women.

**Conclusions:**

Findings support the need to develop and test AOD risk reduction interventions for women from both ethnic groups. In addition, findings point to the need for tailored interventions that target the distinct profiles of AOD use and AOD-related sex risks for HIV among Black African and Coloured women.

## Background

HIV infection is arguably the single greatest threat to public health in South Africa, particularly for women who remain disproportionately affected by the disease [1]. While HIV prevalence in the Western Cape Province is significantly lower than the national estimate, the Western Cape is one of the few provinces where the prevalence of HIV appears to be increasing [2]. As unprotected heterosexual contact is the primary mode of HIV transmission among South African women [2,3], HIV prevention efforts in the country have focused on mass screening for HIV and education about the importance of knowing one’s HIV status and using condoms at every sexual encounter [4,5]. However, these prevention efforts fail to address one of the major contributors to South African women’s vulnerability to HIV, namely alcohol and other drug (AOD) use. AOD use increases vulnerability to HIV largely through its associations with sexual risk taking and also through weakening the immune system [6,7]. Specifically, AOD use leads to disinhibition and impaired judgment that may result in inconsistent condom use and other risky sex behaviors [6-8]. In addition, vulnerable AOD-using women may engage in unsafe sex practices, such as trading sex in exchange for AODs (or money to buy AODs) or they may use AODs to cope with sex trading [9-11] which in itself holds multiple risks for exposure to HIV [10,11].

The failure to address AOD use as part of HIV prevention efforts is of particular concern for women residing in the Western Cape where there are high levels of AOD use [12-16]. Given the high rates of AOD use in the province and evidence of associations between AOD use and sexual risks for HIV, adequate responses to HIV in the Western Cape should include prevention interventions that target AOD-related sexual risk behaviors. Yet, as HIV is not evenly dispersed across all population groups in the Western Cape [1,2,17], it is a more efficient use of scarce resources to focus such interventions on population groups where AOD use is prevalent and HIV incidence is on the rise. In the Cape Town metropolitan area, HIV prevalence and incidence is highest in the predominantly Black African^a^ health districts of Khayelitsha and Gugulethu and second highest in the predominantly Coloured health district of Mitchell’s Plain [17]. These health districts account for the vast majority of the Metro South-East region of the Cape Town metropole which encompasses several large Black African and Coloured communities [18]. Also, the Metro South-East district is disproportionately affected by AOD use, with the bulk of AOD-related treatment admissions in the province stemming from this area [14]. The concentration of AOD use and HIV incident cases in this part of the metropole clearly reflect the need for interventions to reduce AOD-related risks for HIV among Black African and Coloured women residing in this region.

However differences in the health risk profiles of poor Black African and Coloured women are likely given South Africa’s socio-political history of inequality and racial segregation. During apartheid, ethnicity was a major determinant of access to health and other public services, with White South Africans having more access to public services than Coloured South Africans and Coloured citizens having marginally better access to services than Black African South Africans [19-21]. These disparities arose from the legislated geographic segregation of race groups and the distribution of resources along racial lines. Geographical apartheid forced Black African and Coloured South Africans to live in (separate) township areas with limited resources and economic opportunities that were located considerable distances from the well-resourced urban areas reserved for White South Africans [20]. This contributed to greater poverty in township areas which, together with poor access to health services, underpinned ethnic disparities in health risk profiles and disease prevalence [19-21]. Despite 18 years of democracy, South Africa is still grappling with the legacy of apartheid and, for the most part, Black African and Coloured South Africans continue to reside in underserved township areas that, for the most part, remain racially segregated [19]. Ethnicity thus remains an important marker of socio-economic advantage and an important determinant of health in South Africa [19,20]. Consequently, it is important to consider ethnic differences in AOD and HIV-risk profiles when designing AOD-related sexual risk reduction interventions for vulnerable women.

Although earlier work identified differences in AOD use patterns and sexual risk behaviors between Black African and Coloured women [22,23], this work was conducted before the use of methamphetamine became entrenched in the Metro South-East district [14,15,24]. As these prior studies were conducted close to ten years ago, it is quite possible that these ethnic differences in AOD use and sexual risk profiles may have changed. The lack of current knowledge on ethnic differences in AOD-related sexual risk behaviors is cause for concern as it restricts the extent to which HIV risk reduction interventions can be designed to target the different risk profiles of Black African and Coloured women. This paper aims to address this gap through describing differences in AOD use and AOD-related risks for HIV among vulnerable Black African and Coloured women in Cape Town, South Africa.

## Methods

### Study design and participants

This article reports on the baseline characteristics of 720 (324 Black African, 396 Coloured) women recruited into a community-based randomized controlled trial to test an HIV risk-reduction intervention for AOD-using women (the Western Cape Women’s Health CoOp) between September 2008 and January 2011. Participants from other ethnic groups were not recruited into the trial. The details of this study are described elsewhere [25,26].

Eligible participants were women between 18 and 33 years of age, living in one of the study’s target communities who had used at least two drugs (one of which could be alcohol) at least once a week for the past 3 months, had been sexually active with a man in the past month, and had not participated in the previous Women’s Health CoOp pilot or formative studies [22]. To be selected as a target community, areas had to be located within the Metro South-East district of Cape Town and had to be defined as disadvantaged (that is low-income areas designated as Black African or Coloured townships during the apartheid regime) [18]. We sampled across all disadvantaged communities in this region. To ensure balanced recruitment across these communities, population estimates were used to calculate desired sampling targets for each of the selected areas.

### Data collection

Trained outreach workers used standard street-outreach techniques (such as marketing in areas frequented by the target population) to identify potential participants. They approached potential participants and requested verbal permission to administer a brief screening instrument to determine whether they met study eligibility criteria [25,26]. Of the 1098 women who were assessed for eligibility, 247 were excluded because they did not meet eligibility criteria and 131 refused to participate. The main reasons given for refusal to participate were not having time to participate, being suspicious that field personnel were social workers, and not thinking that they had an AOD problem. If eligible women were interested in the study, they were given an appointment for an intake interview, where they were rescreened and enrolled in the trial after providing informed consent. Once enrolled, an interviewer administered the baseline questionnaire by using computer-assisted personal interviewing (CAPI), followed by biological testing for pregnancy, HIV, and the recent use of alcohol, cocaine, methamphetamine, cannabis, opiates, and Mandrax (methaqualone). Participants were provided with refreshments and a grocery voucher valued at ZAR 40 (USD 5.71) for their time. Ethical approval was granted by the Institutional Review Boards of RTI International and Stellenbosch University’s Faculty of Health Sciences (Trial Registration Number: NCT00729391).

### Measures

The baseline questionnaire was a modified Revised Risk Behavior Assessment (RRBA) which was adapted for use in the Western Cape [27]. The RRBA collects self-report information from various domains including demographics and social characteristics, health knowledge, AOD use, sexual risk behaviors, power and empowerment, conflict and victimization (including intimate partner violence), physical and mental health, and the need for services. For this paper, we report on socio-demographic, AOD use, and sexual risk behavior variables only.

#### Socio-demographic variables

Ethnicity was the main independent variable of interest for this paper. For this variable, participants self-identified as Black African or Coloured. In addition, age, education (whether or not they had completed high school), unemployment status (yes/no), and average monthly income (in ZAR) were included in the analyses as potential confounding variables.

#### Alcohol and other drug use

Three of the outcome variables of interest for this paper were self-reported heavy drinking, and recent cannabis and methamphetamine use. These were selected as outcome variables as alcohol, cannabis and methamphetamine are the three most frequently abused substances in the Western Cape Province [14,15]. For alcohol use, we examined a self-report item on alcohol use that explored heavy drinking. Specifically, participants were asked about the number of drinks they consumed on a typical day when drinking. This item had categorical response options with heavy drinking defined as seven or more drinks per day (yes/no).

All participants provided a urine specimen that was tested for the recent use of methamphetamine cocaine, opiates, and THC (cannabis) using the Reditest drug test (Redwood Toxicology Laboratory). Urine was also tested for methaqualone using standard gas chromatography techniques. Results from each of these drug tests were coded as positive (indicating recent use) or negative. For this paper, we only report on the results for cannabis and methamphetamine use.

Items exploring participant’s perceptions of their main sexual partner’s (MSP) AOD use were included in the analyses. Specifically, participants were asked about whether their MSP was drunk or used drugs in the month preceding the study (these items had yes/no response options). Finally, two items examined whether participants thought they had an alcohol or drug problem. This was used as a proxy indicator for treatment need. These items had yes/no response options.

#### Sex risk behavior

Two outcome variables of interest for this study was whether participants’ last sex act was AOD-impaired and unprotected (yes/no), as well as whether their partner was AOD-impaired during their last sex act. AOD-impaired sex was defined as any AOD use just before or during sex.

### Data analysis

All statistics were conducted using SPSS 19. Chi-square tests of association were performed to identify significant associations between ethnicity and categorical socio-demographic, AOD use and sex risk variables. Independent sample *t*-tests were used to compare Black African and Coloured participants on continuously scaled socio-demographic, AOD use and sex risk variables. Five multivariate logistic regression analyses were performed to examine the impact of ethnicity on heavy drinking, current cannabis use, current methamphetamine use, participant AOD-impaired and unprotected at last sex, and partner-impaired at last sex while adjusting for the potentially confounding effects of age, income, education and unemployment.

## Results

### Bivariate analyses

#### Socio-demographic variables

In this sample, Black African women were significantly younger (p <0.0001; Table 1) and had a significantly lower monthly income than Coloured women (p <0.0001). The proportion of participants reporting that they had not completed high school was high (88.89%), with a greater proportion of Coloured participants having not completed high school compared to Black African participants (93.94% versus 82.72%; p <0.0001). Similarly, the proportion of participants reporting that they were unemployed was high (90.00%); with a greater proportion of Coloured participants reporting unemployment compared to their Black African counterparts (92.93% versus 86.42%; p =0.004).

**Table 1:** Sociodemographic, AOD use and AOD-related sex risk variables by ethnicity (N = 720)

**Measure**	**Total Sample (*N *= 720)**	**Black African Women (*n *=324)**	**Coloured Women (*n *=396)**	**Test statistic [t(*df*) or χ**^**2**^**(*df*)]**	***p***
***Sociodemographic variables***					
Age	23.13 (4.25)	22.26 (4.07)	23.85 (4.26)	5.09 (718)	<0.0001
Average income in past month (M, SD)^a^	268.45 (464.03)	168.10 (353.90)	350.60 (523.90)	−5.55 (694)	<0.0001
High school education incomplete (Yes)	640 (88.89%)	268 (82.72%)	372 (93.94%)	22.73 (1)	<0.0001
Unemployed (Yes)	648 (90.00%)	280 (86.42%)	368 (92.93%)	8.39 (1)	0.004
***AOD use variables***					
Past month heavy drinking (Yes)	270 (37.50)	98 (30.25)	172 (43.43)	13.22 (1)	0.0003
Methamphetamine- positive (Yes; n, %)	445 (61.89)	118 (36.53)	327 (82.58)	159.90 (1)	<0.0001
Cannabis-positive (Yes; n, %)	567 (78.86)	303 (93.81)	264 (66.67)	78.61(1)	<0.0001
MSP drunk in past month (Yes)	429 (62.35)	236 (72.84)	193 (53.02)	28.68 (1)	<0.0001
MSP drug use in past month (Yes)	441 (64.10)	162 (50.00)	279 (76.65)	52.90 (1)	<0.0001
Think you have an alcohol problem (Yes)	91 (12.66)	54 (16.67)	37 (9.37)	8.58 (1)	0.003
Think you have a drug problem (Yes)	466 (64.72)	174 (53.70)	292 (73.74)	31.33 (1)	<0.0001
***AOD-related sex risks***					
AOD-impaired and unprotected last sex	276 (38.33)	63 (19.44)	213 (53.79)	88.91 (1)	<0.0001
MSP AOD-impaired at last sex	342 (47.50)	102 (31.48)	240 (60.61)	60.61(1)	<0.0001

^a^Monthly income in ZAR.

#### AOD use variables

Overall, 37.50% of the sample reported past month heavy episodic drinking (Table 1). Coloured women were significantly more likely to report heavy episodic drinking than Black African women (43.43% versus 30.25%; p = 0.0003; Table 1). While close to 80% of the total sample tested positive for recent cannabis use, a significantly greater proportion of Black African women tested positive for cannabis use (93.81%) compared to Coloured women (66.67%; p <0.0001). In addition, while almost two-thirds of the sample tested positive for recent methamphetamine use, ethnic differences in biological testing for methamphetamine were observed (Table 1). Specifically, a greater proportion of Coloured women tested positive for methamphetamine (82.58% versus 36.53%, p<0.0001) than Black African women.

Almost two-thirds of the overall sample reported that their MSP had been drunk in the past month and a similar but greater proportion reported that their MSP had used drugs in the past month. Ethnic differences were also observed for these variables. A greater proportion of Black African participants reported that their MSP had been drunk in the past month compared with Coloured participants (72.84% versus 53.02%; p<0.0001). In contrast, a greater proportion of Coloured participants reported that their MSP had used drugs in the past month compared with Black African participants (76.65% versus 50.00%; p<0.0001).

In terms of treatment need, overall only 12.66% of participants believed they had an alcohol problem. A greater proportion of Black African participants believed they had problems related to the use of alcohol compared to Coloured participants (16.67% versus 9.37%; p = 0.003). While almost two-thirds of the sample believed they had a drug problem, a significantly larger proportion of Coloured women thought they had a drug problem compared to their Black African counterparts (73.74% versus 53.70%; p < 0.0001).

#### AOD-related sexual risk variables

Just over a third of the sample reported being AOD-impaired and having unprotected sex during their last sexual encounter (Table 1). Ethnic differences were observed for these variables, with a greater proportion of Coloured participants reporting being AOD-impaired and having unprotected sex (53.79% versus 19.44%; p <0.0001) than Black African participants. Similarly almost half of the sample reported that their sex partner had used AODs before or during their last sexual encounter. A significantly greater proportion of Coloured women reported that their partner used AODs during their last sex act (60.61% versus 31.48%; p<0.0001) than Black African women (Table 1).

### Multivariate analyses

#### AOD use outcomes

Ethnicity was significantly associated with heavy episodic drinking (Wald Χ^2^ = 8.10 (1), p = 0.004), being cannabis-positive (Wald Χ^2^ = 53.11 (1), p < 0.001), and being methamphetamine-positive (Wald Χ^2^ = 128.69 (1), p < 0.001) even after adjusting for the potentially confounding influences of age, education, income and unemployment in the multiple logistic regression models (Table 2). For the “heavy episodic drinking” model, Coloured women had significantly greater odds of reporting heavy episodic drinking (AOR 1.61; CI: 1.16-2.23) than Black African women. Similarly, for the “methamphetamine-positive” regression model, the odds of testing positive for methamphetamine were more than eight-fold greater for Coloured women than Black African women (AOR 8.43; CI: 5.83-12.18). For the “cannabis-positive” regression model, Coloured women had significantly reduced odds of testing positive for cannabis (by 86%) compared to Black African participants (AOR 0.14; CI: 0.08-0.24; Table 2).

**Table 2:** Logistic regression analyses of ethnicity on AOD use outcomes

**AOD use outcome**	**Covariate**	**Wald test statistic**	***p***	**AOR**^**a**^	**95% CI**^**b**^
***Heavy episodic drinking (yes)***	Ethnicity (Coloured)	8.10	0.004	1.61	1.16–2.23
	Age	0.03	0.860	1.00	0.97–1.04
	Income	3.78	0.052	1.00	1.00–1.00
	Unemployment (No)	6.87	0.009	0.44	0.24–0.82
	Education (Not complete high school)	0.20	0.656	0.89	0.53–1.49
***Cannabis -positive (yes)***	Ethnicity (Coloured)	53.11	<0.001	0.14	0.08–0.24
	Age	3.20	0.074	0.96	0.92–1.00
	Income	8.32	0.004	1.00	1.00–1.00
	Unemployment (No)	0.01	0.937	1.03	0.50–2.11
	Education (Not complete high school)	2.34	0.126	1.68	0.86–3.28
***Methamphetamines -positive (yes)***	Ethnicity (Coloured)	128.69	<0.001	8.43	5.83–12.18
	Age	0.00	0.997	1.00	0.96–1.04
	Income	0.42	0.515	1.00	1.00–1.00
	Unemployment (No)	5.97	0.015	0.48	0.27–0.86
	Education (Not complete high school)	4.59	0.032	0.54	0.31–0.95

^a^ AOR = adjusted odds ratio.

^b^ 95% CI = 95% confidence intervals.

#### AOD-related sex risk outcomes

Ethnicity was significantly associated with being AOD-impaired and unprotected at the last sex encounter (Wald Χ^2^ = 60.24 (1), p < 0.001) and MSP being AOD-impaired at the last sex encounter (Wald Χ^2^ = 47.64 (1), p < 0.001) even after adjusting for age, education, income and unemployment in the logistic regression models (Table 3). For the “AOD-impaired and unprotected at last sex” regression model, Coloured participants had four-fold greater odds of reporting that their last sexual encounter was AOD-impaired and unprotected (AOR 4.05; CI: 2.85-5.77) than Black African participants. Similarly, for the “MSP AOD-impaired at last sex” regression model, Coloured women had three-fold greater odds of reporting that their MSP was AOD-impaired at last sex (AOR 3.16; CI: 2.28-4.38) than Black African participants.

**Table 3:** Logistic regression analyses of ethnicity on AOD-related sex risk outcomes

**AOD use outcome**	**Covariate**	**Wald test statistic**	***p***	**AOR**^**a**^	**95% CI**^**b**^
***AOD impaired and unprotected at last sex (yes)***	Ethnicity (Coloured)	60.24	<0.001	4.05	2.85–5.77
	Age	6.07	0.014	1.05	1.01–1.09
	Income	6.52	0.011	1.00	1.00–1.00
	Unemployment (No)	1.12	0.290	0.72	0.39–1.33
	Education (Not complete high school)	0.33	0.568	1.18	0.67–2.09
***MSP AOD-impaired at last sex (yes)***	Ethnicity (Coloured)	47.64	<0.001	3.16	2.28–4.38
	Age	0.16	0.692	1.01	0.97–1.05
	Income	1.91	0.167	1.00	1.00–1.00
	Unemployment (No)	3.69	0.065	0.58	0.33–1.01
	Education (Not complete high school)	0.77	0.382	0.80	0.48–1.33

^a^ AOR = adjusted odds ratio.

^b^ 95% CI = 95% confidence intervals.

## Discussion

Our findings have several implications for the design and implementation of behavioral interventions to reduce AOD use and AOD-related sex risks for HIV among vulnerable women in South Africa. First, findings highlight the need for AOD use interventions among vulnerable women, irrespective of ethnicity. However, findings also indicate that Black African and Coloured women have different profiles of AOD use. Specifically, we found that Coloured women had significantly greater odds of testing positive for methamphetamine and self-reporting heavy episodic alcohol use and significantly reduced odds of testing positive for cannabis than Black African women. These findings suggest that while Black African women are more likely to use cannabis than Coloured women, they are less likely to have problematic AOD use (as indicated by the pervasiveness of methamphetamine use and heavy episodic drinking among Coloured women in this sample). This is supported by the finding that Coloured participants in our study were also significantly more likely to report that they believed they had a drug problem compared to their Black African counterparts. These findings are broadly in keeping with those from earlier studies that reported less AOD use (with the exception of cannabis) among women from Black African communities relative to women from Coloured communities in the Western Cape [22,23].

Continuing ethnic disparities in access to income provide a possible explanation for these different profiles of AOD use, with Coloured persons being marginally better off and therefore potentially more able to afford AODs than Black African citizens [19-21]. Yet differences in income do not account for our findings. Even after controlling for the potentially confounding effects of age income, education and employment status, ethnic differences in AOD use outcomes remain. It is possible that contextual (such as drug marketing practices [28]) and cultural differences between Black African and Coloured communities may contribute to these unique profiles of AOD use, however this requires further investigation. Regardless of the reasons for these differences, our findings imply that AOD use interventions should be tailored to the distinct profiles of AOD use among Black African and Coloured women. Our findings suggest that AOD interventions for vulnerable Coloured women should focus on reducing heavy episodic drinking and providing treatment for methamphetamine use. While there are several drug treatment programs that address methamphetamine-related problems in the Western Cape, vulnerable women face more affordability and geographic access barriers to utilizing these services than men [29]. The high prevalence of methamphetamine use and self-identified drug problems among Coloured women in our sample indicate an urgent need to address these barriers to treatment through introducing (where absent) and scaling up (where available) community-based AOD treatment services that are affordable and easily accessible for vulnerable Coloured women.

In contrast, our findings suggest that AOD interventions for vulnerable Black African women should consist mainly of low-intensity, brief interventions that focus on reducing cannabis use and educating women about the risks associated with methamphetamine and other drug use. As several studies have shown that frequent and regular use of cannabis is associated with increased risk of transition to other illicit drug use [30,31], providing Black African women with brief interventions focused on reducing cannabis use may not only help them reduce the negative consequences associated with their cannabis use, but may also prevent progression from cannabis to methamphetamine use.

However, this does not mean that AOD interventions focused on reducing methamphetamine use are not needed in Black African communities. Ensuring that Black African women have access to interventions for methamphetamine use is essential given that more than a third of Black African participants tested positive for the recent use of methamphetamine. This unexpected finding is in sharp contrast to earlier work which has consistently pointed to low rates of methamphetamine use among Black African populations in Cape Town [22,32,33]. This sudden increase in the use of methamphetamine among Black African women points to the urgent need for AOD prevention interventions that address the underlying risk factors that predispose Black African women to initiate methamphetamine use as well as treatment interventions that address risk factors for the continued use of methamphetamine. These interventions, combined with other interventions that address environmental and structural risk factors for drug use [34], may help curtail the further escalation of methamphetamine use within Black African communities.

Failure to provide and, where available, scale up AOD interventions for vulnerable Black African and Coloured women not only represents a missed opportunity to intervene with their AOD use but may have unintended negative consequences for efforts to curb the spread of HIV in the region. This study found that AOD-impaired sex is widespread among vulnerable AOD-using women, with close to 40% of our sample reporting that their last sexual encounter was not only AOD-impaired but also unprotected. Compared to Black African participants, Coloured participants had four-fold greater odds of reporting that their last sexual encounter was AOD-impaired and unprotected, even after adjusting for age, education, income and employment status. One explanation for this finding is that AOD use before or during sex reduces personal perceptions of risk for HIV thus leading to less condom use [35]. Personal perceptions of risk for HIV might already be lower among Coloured women than among Black African women because of the lower prevalence of HIV in Coloured communities [2,17]. This explanation is supported by prior studies conducted in this region that identified a negative relationship between HIV risk perception and engagement in sex risk behaviors for women [36]. Regardless of the reason for these findings, they clearly point to the urgent need for behavioral interventions that focus on reducing AOD use before or during sex and reducing other barriers to condom use among vulnerable women in Cape Town. While findings suggest that vulnerable women from both ethnic groups would benefit from AOD-related sex risk reduction interventions, the need seems particularly great among women from Coloured communities where AOD-impaired sex is prevalent and condom use low.

Third, our findings suggest that for AOD-related sex risk reduction interventions to be impactful, such interventions should target AOD use among vulnerable women as well as their main sexual partners. Close to two-thirds of our sample reported that their main sexual partner had either been drunk or had used drugs during the month preceding the study and almost one in two participants reported that their main sexual partner had been AOD-impaired during their last sexual encounter. This is cause for concern as evidence from earlier studies suggests that women with partners who use AODs have a poorer response to AOD interventions than women with partners who do not use AODs [37]. In addition, the likelihood of implementing sex risk reduction strategies (such as consistent condom use) within relationships is diminished if one or both partners are AOD-impaired during sex [38,39]. Finally, as we found ethnic differences in partner-related AOD-impaired sex (with Coloured women having three-fold greater odds of reporting that their main partner was AOD-impaired at last sex relative to Black African women), the need for these proposed interventions seems most pressing among Coloured women and their main sexual partners.

Despite the important implications that these findings have for HIV prevention programming, these results should be considered in the light of some methodological limitations. First, this paper was based on the analysis of baseline data from a larger HIV and AOD risk reduction project. The strict inclusion and exclusion criteria of this study may have adversely impacted the ability to recruit a representative sample of Black African and Coloured South African women. Second, as the sample was recruited from poor communities within one region of the Cape Town metropole, the extent to which findings can be extrapolated to other parts of the province or country are unclear. Third, in this study women provided self-report data on their main sexual partner’s AOD use. These perceptions are open to several reporting biases and it is quite possible that social desirability and other processes may have affected the accuracy of the information provided. This last limitation highlights the need for future work among vulnerable women and their main sexual partners so that AOD use and related sexual risk behaviors that occur within the context of relationships can be further explored.

## Conclusions

This paper provides evidence of ethnic differences in AOD use patterns and AOD-related sex risks for HIV among vulnerable women in Cape Town, South Africa. Findings suggest that Coloured women have more entrenched AOD problems, particularly related to the use of methamphetamine, and are more likely to report AOD-related sex risks than Black African women. Findings suggest that while vulnerable women from both ethnic groups would benefit from behavioral interventions to reduce AOD use and AOD-related sex risk behaviors, tailored interventions are needed that target the unique profiles of AOD use and AOD-related sex risks for HIV among Black African and Coloured women.

## Endnotes

^a^The terms “White, Black African, Asian/Indian, and Coloured” refer to demographic markers that were chosen for their historical significance and are still used today. “Coloured” refers to a grouping of people of mixed race ancestry that self-identify as a particular ethnic and cultural grouping in South Africa. The continued use of these markers is important for identifying ethnic disparities in health and for monitoring improvements in health and socio-economic disparities in South Africa.

## Abbreviations

AOD: Alcohol and other drug;HIV: Human immunodeficiency virus;CAPI: Computer-assisted personal interview;RRBA: Revised risk behavior assessment;MSP: Main sexual partner

## Competing interests

The authors declare that they have no competing interests.

## Authors’ contributions

BM was a co-investigator on the study and was responsible for planning, writing and reviewing all aspects of the manuscript. TLK conducted the statistical analyses with inputs from BM and WMW and assisted with writing the methods. FAB, KJ, TC and CP reviewed the draft manuscript and provided critical comments. WMW is the principal investigator for this project and takes responsibility for the integrity of the data and the accuracy of the data analysis. She is the senior author on this paper and provided inputs into every aspect of the paper. All authors read and approved the final draft.

## Pre-publication history

The pre-publication history for this paper can be accessed here:

http://www.biomedcentral.com/1471-2458/13/174/prepub
